# Distance Estimation in Virtual Reality Is Affected by Both the Virtual and the Real-World Environments

**DOI:** 10.1177/20416695211023956

**Published:** 2021-06-18

**Authors:** Junjun Zhang, Xiaoyan Yang, Zhenlan Jin, Ling Li

**Affiliations:** MOE Key Lab for Neuroinformation, The Clinical Hospital of Chengdu Brain Science Institute, 12599 University of Electronic Science and Technology of China, Chengdu, China

**Keywords:** virtual reality, egocentric distance perception, environmental context

## Abstract

The experience in virtual reality (VR) is unique, in that observers are in a real-world location while browsing through a virtual scene. Previous studies have investigated the effect of the virtual environment on distance estimation. However, it is unclear how the real-world environment influences distance estimation in VR. Here, we measured the distance estimation using a bisection (Experiment 1) and a blind-walking (Experiments 2 and 3) method. Participants performed distance judgments in VR, which rendered either virtual indoor or outdoor scenes. Experiments were also carried out in either real-world indoor or outdoor locations. In the bisection experiment, judged distance in virtual outdoor was greater than that in virtual indoor. However, the real-world environment had no impact on distance judgment estimated by bisection. In the blind-walking experiment, judged distance in real-world outdoor was greater than that in real-world indoor. On the other hand, the virtual environment had no impact on distance judgment estimated by blind-walking. Generally, our results suggest that both the virtual and real-world environments have an impact on distance judgment in VR. Especially, the real-world environment where a person is physically located during a VR experience influences the person’s distance estimation in VR.

One important goal of virtual reality (VR) technology is to make the experience in the virtual environment mimic that of the real environment. Egocentric distance perception is one of the fundamental measurements of the visual space (Loomis & Knapp, 2003; Renner et al., 2013). Veridical distance perception in VR is important to applications (Renner et al., 2013) such as military training. Thus, distance perception in the VR environment has been studied extensively (Bruder et al., 2015; Creem-Regehr et al., 2015; Kelly et al., 2017; Kunz et al., 2015; for a review, see Renner et al., 2013).

One factor that has been found to have an impact on distance perception is the environmental context. The effect of environmental context has been observed in studies performed in real-world (Bodenheimer et al., 2007; Iosa et al., 2012; Lappin et al., 2006; Philbeck et al., 2018; Witt et al., 2007), in augmented reality (Livingston et al., 2009), and in VR environment (Kelly et al., 2017, 2018). The previous studies have demonstrated that the perceived distance is influenced not only by the physical dimensions of the environmental layout (Lappin et al., 2006; Witt et al., 2007) but also by observers’ knowledge of the layout, which can be acquired through previews (Kelly et al., 2018) and interactions (Kelly et al., 2018; Philbeck et al., 2018). In VR, the observer is located in a real world that is usually different from the virtual scene, resulting in a perceptual dissociation between the real-world and the virtual environment. Under such circumstances, observers may have a completely immersed experience in the VR environment or may still feel somewhat in the real-world environment. Presumably, distance perception can be influenced by both the real-world and the VR environment. The more immersive experience the observer has in VR, the more likely it is that the perceived distance will be affected by the VR environment. On the other hand, if the observer is aware of the real world, the real-world environment may also have an impact on the perceived distance.

The current study aimed to investigate how environmental context influences distance perception, using a VR head-mounted display (HMD). We proposed that environmental context has two components: the real-world and the virtual environment. Specifically, we tested the influence of the real-world environment, when observers were aware of their real-world surroundings during the VR experience. Previous studies have compared the distance estimation in different VR environments (Creem-Regehr et al., 2015) when observers were located in only one real environment. However, no one has tested whether, and to what extent, would two different real-world environments have an impact on the VR experience. In the present study, observers were asked to perform an identical VR distance judgment task, when they were in two different real environments. This differed from the previous studies, where observers were blindfolded before entering either the experimental environment (Creem-Regehr et al., 2015; Geuss et al., 2012) or the real-world environment kept constant through the experiment (Interrante et al., 2006; Kelly et al., 2018). In the present study, subjects were always aware of the different real-world environments used during the experiment. This is important because, in some of the VR applications, users are aware of their surroundings. Therefore, if such awareness might affect the distance estimation in VR, such a distortion needs to be acknowledged.

There are different methods to measure distance perception (Loomis & Philbeck, 2008), including the categorization of verbal estimates, perceptual matching, and visual directed actions (Renner et al., 2013). Usually, the distance range was within action space (Cutting & Vishton, 1995), from around 2 m to 25 m from the observers. One of the most commonly used methods is blind-walking, whereby participants view a target and are then blindfolded and asked to walk toward the estimated location of the target. Studies have shown that in real-world environments, under full-cue viewing conditions, there is no systematic error when performing a blind-walk from around 1–2 m to 20–25 m on the ground (Loomis & Knapp, 2003; Sinai et al., 1998; Wu et al., 2004), while other studies have observed a small yet consistent underestimation of the egocentric distance perception (Bian & Andersen, 2013; Gajewski et al., 2010, 2014; Z. Li et al., 2011). On the other hand, in VR environments, the distance was underestimated in blind-walking tasks (Loomis & Knapp, 2003; Messing & Durgin, 2005), at a mean rate of 74% of the veridical distance (Renner et al., 2013), especially with HMD systems (Plumert et al., 2005). However, more recent studies have shown that the perceived egocentric distance, using advanced HMD systems, is more accurate and closer to that in real world (Buck et al., 2018; Kelly et al., 2017; B. Li et al., 2016).

Another method that is often used to measure distance perception is perceptual matching, which, unlike blind-walking, does not involve motor responses (Philbeck & Loomis, 1997). Bisection is one of the methods of perceptual matching. Bisection requires a participant to determine the midpoint of a distance interval between themselves and a target. In a real-world environment, bisection is as accurate as blind-walking (Rieser et al., 1990). A study by Bodenheimer et al. (2007), using the bisection method, found that accurately estimated distance in the real world was nonlinearly compressed in VR. That is, the estimated midpoint distance increased at a rate lower than the actual midpoint distance did. Note that the distance estimation may vary according to the measurement method. While absolute distance is estimated in blind-walking, in the bisection method, the estimation depends on the relative distance as well. Thus, perhaps it is not appropriate to compare the measured distance directly between these two methods under the same environmental context. However, it is still possible to investigate the influence of environmental context separately, for blind-walking and bisection.

In the present study, we used these two measurement methods of distance perception—bisection and blind-walking. We hypothesized that environmental context would have a differential effect on the two methods. Because bisection is a closed-loop method where visual feedback in the virtual scene is given when the judgment is made, the virtual environment would affect distance estimation. On the other hand, with blind-walking, observers are required to walk in the real world with no visual feedback from the virtual scene, and in this case, the real-world environment would affect distance estimation. We used constant error (CE, aka systematic error) to measure the accuracy of the judged distance and variable error (VE, aka random error) to measure the precision. Specifically, CE measured the systematic bias of the judged distance, that is, whether the distance was underestimated, overestimated, or correctly estimated. On the other hand, VEs measured the consistency of the judgments, that is, the variations across trials.

To test the previous hypotheses, we performed three experiments: In Experiment 1, we used the bisection method, while we used the blind-walk method in Experiments 2 and 3. All the experiments were carried out in two locations on our campus—one was an indoor hallway and the other was an outdoor pathway. Replicas of the two locations were installed in VR. Moreover, distance estimation was evaluated for the following four combined conditions: the real hallway location with the virtual hallway scene, the real hallway location with the virtual pathway scene, the real pathway location with the virtual hallway scene, and the real pathway location with the virtual pathway scene.

## Experiment 1

### Methods and Materials

#### Apparatus

Different virtual environments were displayed using an HTC VIVE headset, and headset displays were generated using Unity3D with NVidia GeForce GTX 1070 graphics card. The headset displays had a resolution of 1,080 × 1,200, a field of view (FOV) of 110° diagonally per eye, and a refresh rate of 60 Hz. The position and orientation were tracked in three dimensions using the Lighthouse tracking system.

#### Stimuli

**Figure 1.**
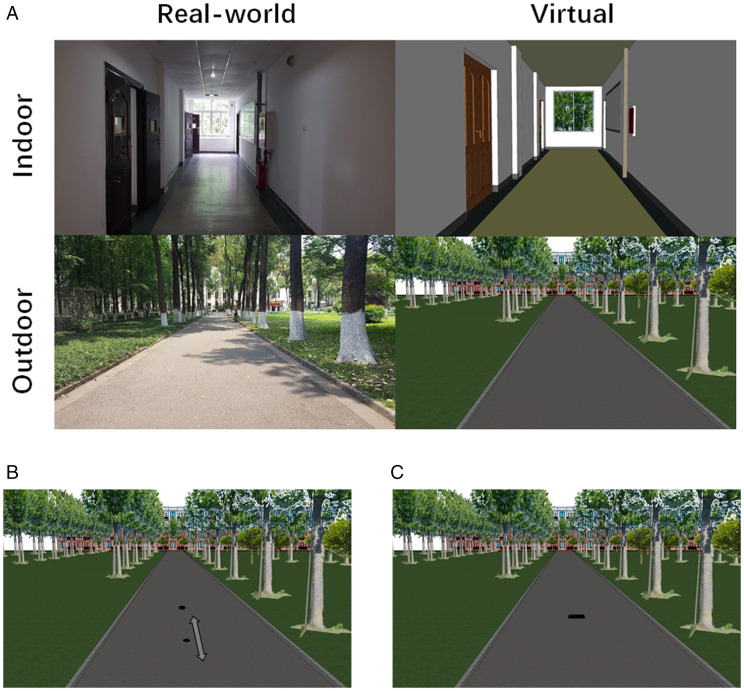
(A) The photos of the two real-world locations where the experiments were taken place and the models of the two locations in the virtual scenes. The replicas in the VR simulated the metric information of the real world (such as the extensions of the scenes and the locations of the trees and doors) but did not simulate all its details. (B) The bisection method in Experiment 1. Observers were asked to adjust the position of the nearest target in depth to match the midpoint of the farthest target. (C) The blind-walking method in Experiment 2. Observers were asked to view the target first and then walk to the estimated distance blindfolded. Notice that the size of the targets in the figures is for the purpose of demonstration and was not identical in the experiments.

Two real-world environments on our campus—an indoor hallway and an outdoor pathway—were selected as the experiment locations. The replicas of these two environments were modeled into VR, where the experiment was performed (Figure 1A). The distance from the simulated starting position of the participants, to the far end of the terrain, was 14.5 m and 90 m in the virtual indoor and outdoor environments, respectively. The width of the indoor hallway and the outdoor pathway was 2.64 m and 4.67 m, respectively. Note that the targets only appeared in virtual environments. More importantly, the participants were aware of where they really were during the experiment. In VR, the simulated eye-heights for participants were auto-adjusted by the HTC VIVE system according to their real-world eye-heights. Participants wore a pair of earbuds during the whole experiment to prevent ambient noise from the real world.

#### Participants

Twenty-eight college students (age 21–26 years, 10 females) who were naive to this study participated in the experiment. All participants had normal or corrected-to-normal vision and were right-handed. This research complied with the tenets of the Declaration of Helsinki and was conducted in accordance with the recommendations of “the Guideline for Human Behavior Studies, the Institutional Review Board of UESTC MRI Research Center for Brain Research.” Written informed consent was obtained from each participant before enrollment. Finally, the protocol of the present study was approved by the Institutional Review Board of UESTC MRI Research Center for Brain Research.

#### Design

Bisection was used in the current experiment. In each trial, two black disks were presented on the floor right in front of the participant (Figure 1B). The disks were positioned 0.001 m above the ground to generate a perception of cardboard lying on the ground. The position of the farther disk was fixed while the initial position of the nearer disk was placed randomly in depth between the participants and the farthest disk. The participants were asked to move a mouse (Logitech G100s) back and forth to adjust the position of the nearer disk until they felt it was at the midpoint between the participant and the farther disk and then click the left mouse button to confirm the estimation. Note that the adjustment of the position was only along the direction in depth. After distance estimation, both the disks disappeared for 2 seconds before the initiation of the proceeding trial. The radius of the disk was 0.025 m, at a distance of 4 m, corresponding to approximately 0.67 degrees in horizontal angular size. The size of the disk varied according to its distance to the observer, to keep the horizontal angular size constant so that the angular size would not become a cue for the distance judgment.

The distance of the farther disk to the participant was varied across trials, from 4 m to 7.8 m, with an interval of 0.2 m. Thus, there were 20 different distances in total. Within a block, each distance was tested once, and there were 20 trials in each block. Table 1 displays the design of the experiment. Participants were divided into two groups and performed the experiment on 2 days separated by a 7-day interval. Participants in Group 1 performed the first session (Day 1) in the real-world indoor hallway and the second session (Day 2) on the real-world outdoor pathway. Each session consisted of four blocks, and the order of the blocks was counterbalanced with an ABBA design. Within each block, only one virtual environment (indoor hallway or outdoor pathway) was tested. For instance, a participant from Group 1 would be asked to come to the real-world indoor hallway on the first day and to perform four blocks of the bisection method in a virtual “indoor-outdoor-outdoor-indoor” order. After 7 days, the participant would come to the real-world outdoor pathway and then perform four blocks of the bisection method with a virtual “outdoor-indoor-indoor-outdoor” order. Participants in Group 2 performed the same experiment as participants in Group 1, except that they arrived at the real-world outdoor pathway on the first day and at the real-world indoor hallway on the second session (Day 2, 7 days later). Hence, the order of both the real-world and the virtual environments was counterbalanced.

**Table 1.** The Averaged Estimated Midpoint, CE, and VE for Each Condition in Experiment 1.

		Day 1: Real-world indoor		Day 2: Real-world outdoor	
		Virtual indoor	Virtual outdoor	Virtual indoor	Virtual outdoor
Group 1 (*n* = 14)	Estimated midpoint	2.69 m	2.45 m	2.70 m	2.54 m
	Constant error	–8.3%	–16.1%	–7.8%	–12.9%
	Variable error	6.2%	8.2%	6.4%	7.6%
		Day 1: Real-world outdoor		Day 2: Real-world indoor	
		Virtual indoor	Virtual outdoor	Virtual indoor	Virtual outdoor
Group 2 (*n* = 14)	Estimated midpoint	2.80 m	2.68 m	2.76 m	2.62 m
	Constant error	–4.7%	–8.2%	–5.6%	–10.3%
	Variable error	7.0%	8.0%	7.8%	7.4%

### Results

**Figure 2.**
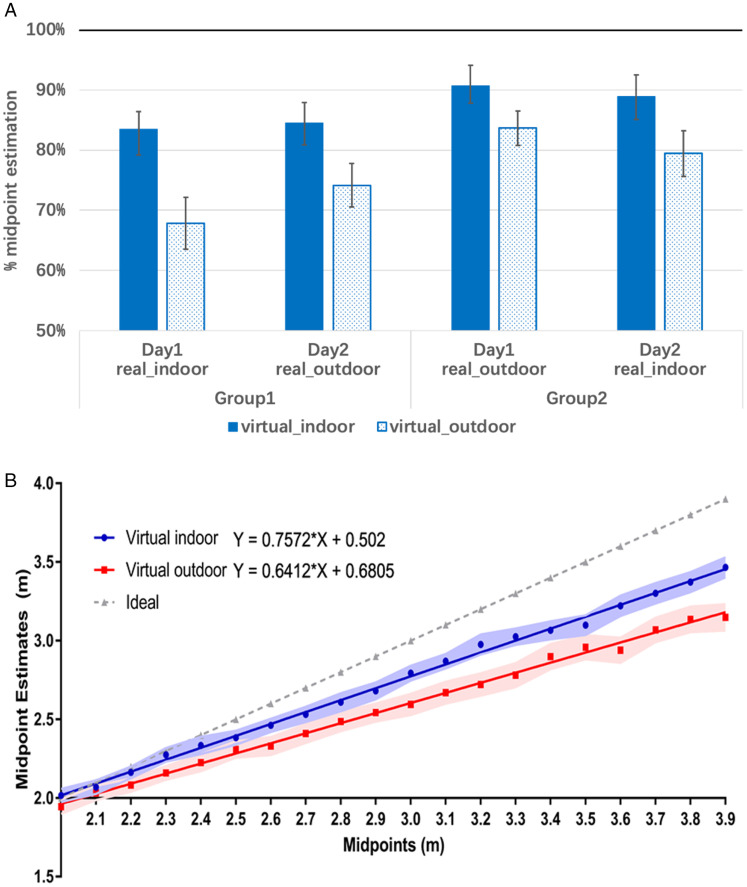
(A) The ratio of the estimated midpoint to the target in Experiment 1. Error bars denote standard error. The ideal performance is 100%. (B) Averaged midpoint estimates across distances in virtual indoor and outdoor environments. The light color band denotes the range of the standard errors.

We evaluated and compared three independent variables: (a) virtual environment (virtual indoor and outdoor), (b) real-world environment (real-world indoor and outdoor), and (c) practicing effect (Day 1 and Day 2). The dependent variable was the mean estimation of the midpoints. The mean estimated midpoints of each condition are presented in Table 1 and Figure 2A. Moreover, we analyzed the CEs and VEs for each condition. For each observer, CE was calculated as the average of the ratio of (judged—veridical midpoint distances) to the veridical midpoint distances, and VE was calculated as the standard deviation of the same ratio. Then, a three-way (virtual environment, real-world environment, and practicing effect) repeated analysis of variance (ANOVA) was applied to compare CEs and VEs. For CEs, the results showed a significant main effect of the virtual environment, *F*(1, 13) = 19.508, *p* < .01, $\eta_{p}^{2}$ =0.600. Particularly, CE in the virtual outdoor environment (–11.9%) was more negative than that in the virtual indoor environment (–6.6%). For VEs, results showed a significant main effect of the virtual environment, *F*(1, 13) = 9.553, *p* < .01, $\eta_{p}^{2}$ =0.424. VE in the virtual outdoor (7.8%) was higher than that in the virtual indoor (6.8%). No other main effects or interactions were found to be significant for either CEs or VEs. CEs and VEs for each condition are displayed in Table 1.

Figure 2B illustrates the estimated midpoints across all the distances in different virtual environments, showing that the estimated midpoint was farther in virtual indoor than virtual outdoor across distances. We further performed a linear regression for both the virtual indoor (Y = 0.7572 × X + 0.5020, *R*^2^ = .9975) and the virtual outdoor (Y = 0.6412 × X + 0.6805, *R*^2^ = .9968). A binomial test was performed to test the difference between the judged and veridical midpoint distances. Results showed that the judged midpoint distances were significantly underestimated in both the virtual outdoor environment (*p* < .001) and the virtual indoor environment (*p* < .05).

To summarize the results of Experiment 1, we found that midpoint distance judgments in VR showed larger underestimations in the virtual outdoor environment, compared with those in the virtual indoor environment. The consistency of the judgments was better in the virtual indoor compared with virtual outdoor. In contrast, the real-world environment did not affect the distance judgments in this experiment.

## Experiment 2

In Experiment 1, we demonstrated that the virtual environment, not the real-world environment, affects distance judgment in VR. The task in Experiment 1 was bisection. In Experiment 2, we tested the distance estimation using blind-walking, which requires walking in the real world.

### Methods and Materials

#### Participants

Forty college students (age 20–25 years, 15 females) who were naive to this study participated in the experiment. All participants had normal or corrected-to-normal vision and were right-handed. None of them had participated in Experiment 1.

#### Design

Blind-walking was used in the current experiment. The apparatus and the stimuli were the same as those in Experiment 1. In each trial, a target was placed on the ground in front of the participant. The distance from the center of the target to the participant varied from 3 m to 5.5 m, with a 0.5 m interval. The target appeared as a black rectangle, which subtended a visual angle of approximately 0.8 degrees (vertically) by 5.0 degrees (horizontally) across different distances. Thus, the size did not provide a cue for the distance. In each trial, participants were asked to wear the HMD and viewed a scene that included the target. After about 5 seconds, participants were asked to close their eyes, remove the HMD, and were blindfolded by eye patches. They were then asked to walk at their normal pace, to the location where the target was presented. While participants were walking, one experimenter walked with the participants to ensure that they walked in the correct direction. Participants were told to stop when they thought they reached the target location. The walked distance was measured using a digital laser rangefinder, and participants were asked to turn around and were led by the experimenter to the original location before continuing with the next trial. Participants opened their eyes only with the HMD on during the experiment.

Participants were divided into four groups, and each of them was asked to come to the experiment twice, with a 7-day interval between the two sessions. Each group consisted of 10 participants. The design of the experiment is shown in Table 2. Groups 1 and 2 viewed the indoor hallway as the virtual environment throughout the experimental sessions. The other participants only viewed the virtual outdoor pathway throughout the experimental sessions. On Day 1 (first session), participants in Group 1 and Group 3 performed the experiment in the real-world indoor hallway, and after 7 days (second session), they performed the experiment in the real-world outdoor pathway. The order of the real-world environments for Group 2 and Group 4 was swapped. In each session, each participant performed the blind-walking method for 15 trials, with 3 repetitions across 5 different distances. The order of the trials was randomized with the constraint that no adjacent trials were of the same distance. Before the experiment, participants were asked to close their eyes and blindly walked back and forth a few times to familiarize themselves with blind-walking.

**Table 2.** The Averaged Estimated Midpoint, CE, and VE for Each Condition in Experiment 2.

		Group 1 (*n* = 10)		Group 2 (*n* = 10)	
		Day 1: Real-world indoor	Day 2: Real-world outdoor	Day 1: Real-world outdoor	Day 2: Real-world indoor
Virtual indoor	Walked distance	3.68 m	4.61 m	3.99 m	4.21 m
	Constant error	–14.0%	7.5%	–7.0%	–1.6%
	Variable error	5.9%	9.2%	8.1%	7.4%
		Group 3 (*n* = 10)		Group 4 (*n* = 10)	
		Day 1: Real-world indoor	Day 2: Real-world outdoor	Day 1: Real-world outdoor	Day 2: Real-world indoor
Virtual outdoor	Walked distance	4.15 m	4.69 m	4.40 m	4.25 m
	Constant error	–2.8%	9.4%	2.6%	–1.2%
	Variable error	7.1%	8.9%	9.7%	8.9%

### Results

**Figure 3.**
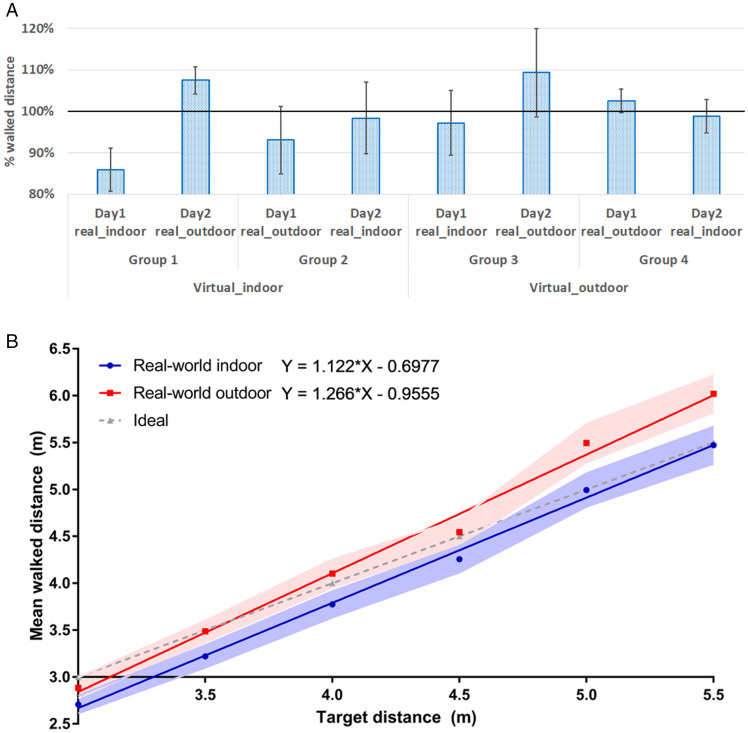
(A) The ratios of the walked distances to the target distances in Experiment 2. Error bars denote standard error. (B) Averaged walked distances at all the distances in real-world indoor and outdoor environments. The light color band denotes the range of the standard errors.

We evaluated and compared three independent variables: (a) virtual environment (virtual indoor and outdoor), (b) real-world environment (real-world indoor and outdoor), and (c) practicing effect (Day 1 and Day 2). The dependent variable was the mean walked distance. The mean walked distances in each condition are illustrated in Table 2 and Figure 3A. We compared the CEs and VEs using a three-way mixed ANOVA. For the CEs, we found significant main effect of the real-world environment, *F*(1, 36) = 10.569, *p* < .01, $\eta_{p}^{2}$ = 0.227, and of the practicing effect, *F*(1, 36) = 12.678, *p* < .01, $\eta_{p}^{2}$ = 0.260. CE in the real-world outdoor environment (3.1%) was greater than that in the real-world indoor environment (–4.9%). CEs on the second day (3.5%) were greater compared to that of the first day (–5.3%). For the VEs, we observed a significant main effect of the real-world environment, *F*(1, 36) = 8.239, *p* < .01, $\eta_{p}^{2}$ = 0.186. VE in the real-world outdoor (9.0%) was greater compared to the real-world indoor (7.3%) environment. No other significant main effects or interactions were found for either CEs or VEs. An illustration of CEs and VEs for each condition is in Table 2.

Figure 3B illustrates the walked distance across all distances, showing that the walked distance was longer in real-world outdoor than in real-world indoor across distances. In addition, we performed a linear regression for both real-world outdoor (Y = 1.266 × X – 0.9555, *R*^2^ = .9922) and real-world indoor (Y = 1.122 × X – 0.6977, *R*^2^ = .9968). A binomial test was applied to investigate the differences between the walked and the veridical distances. However, no significant differences were found for either the real-world indoor or outdoor environment.

To summarize the results of Experiment 2, the walked distances in the real-world outdoor environment were greater than the walked distances in the real-world indoor environment. The consistency of the judgments was better in real-world indoor compared with real-world outdoor Nevertheless, there were no significant differences between the walked and veridical distances. Overall, the virtual environment did not affect the distance judgments in this experiment.

## Experiment 3

In Experiment 2, the virtual environment did not have an impact on the walked distance, possibly because each observer only viewed one virtual scene. Thus, the between-subject comparison may have failed to detect the virtual environmental effect. In this experiment, we analyzed the effect of the virtual environment using the within-subject design that was used in Experiment 1.

### Methods and Materials

#### Participants

Fourteen college students (age 21–23 years, 6 females) who were naive to this study participated in the experiment. All participants had normal or corrected-to-normal vision and were right-handed. None of them had participated in Experiment 1 or Experiment 2.

#### Design

The blind-walking method was the same as in Experiment 2, using the same apparatus and stimuli. The experiment was performed in a real-world hallway. The experiment consisted of four blocks, and the order of the blocks was counterbalanced with an ABBA design. Within each block, only one virtual environment (indoor hallway or outdoor pathway) was tested. In each block, the participants performed the blind-walking method for five trials, across five different distances (3 to 5.5 m with a 0.5 m interval). The order of the virtual scenes was counterbalanced.

#### Bayesian Analysis

It is likely that even with a within-subject design, there is still no significant virtual environment effect on the walked distance, which favors the null hypothesis. However, it is impossible to assess if the data favor the null hypothesis in the classical null hypothesis significance testing framework. Nevertheless, the Bayesian framework can provide evidence for the null hypothesis, by measuring how much more likely the data are under the null hypothesis compared to the alternative hypothesis (Quintana & Williams, 2018). Thus, we also performed a Bayesian paired *t* test with JASP version 0.14.1 (JASP Team, 2020) to test whether the virtual environment has an impact on walked distance. The prior is defined by a zero-centered Cauchy distribution with a scale of 0.707.

### Results

The effect of the virtual environment (indoor vs. outdoor) was evaluated using CEs and VEs. The CEs for virtual indoor and outdoor were –6.1% and –8.1%, respectively. A paired *t* test showed a nonsignificant difference for CE, *t*(_13_) = 0.711, *p* = .490. The VEs for virtual indoor and outdoor were 4.0% and 3.9%, respectively. A paired *t* test showed a nonsignificant difference for VEs, *t*(_13_) = 1.438, *p* = .174. Further, a Bayesian paired sample *t* test was performed to compare CEs indoor and outdoor. The result showed BF_10_ = 0.336, which provides moderate evidence that CEs are more likely to be the same indoor/outdoor than they are different.

**Figure 4.**
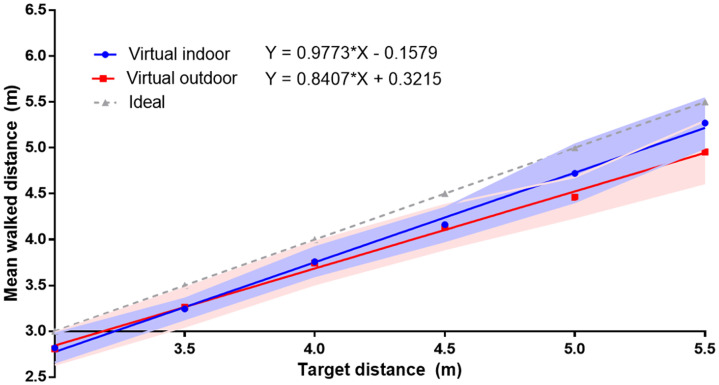
Averaged walked distances at all the distances in real-world indoor and outdoor environments in Experiment 3. The light color band denotes the range of the standard errors.

Figure 4 illustrates the walked distance in virtual indoor and outdoor environments. A binomial test was applied to investigate the differences between the walked and veridical distances. Our analyses showed a tendency for the walked distances to be underestimated in both the virtual indoor (*p* = .057) and the virtual outdoor (*p* = .057) environments.

## Discussion

In this study, we examined distance estimations within the action space using an HMD. Previous studies have investigated the effect of virtual environmental context on distance estimation in VR (Bodenheimer et al., 2007; Kelly et al., 2017, 2018). However, no evidence has shown that the real-world environment, where you actually are during a VR experience, has an impact on distance judgment in VR. Our study, for the first time, has demonstrated that both the real-world and the virtual environment can influence distance judgment in VR. Specifically, with the method of bisection (Experiment 1), judged midpoint distance was closer in the virtual indoor environment than in the virtual outdoor environment. With the method of blind-walking (Experiments 2 and 3), walked distance was farther in the real outdoor environment than that in the real indoor environment.

The main finding of interest in Experiment 1 was that the judged midpoint distance in virtual outdoor was shorter than that in virtual indoor. We propose that these differences may be related to the linear perspective and the terrestrial horizon, which is defined as the visible far end of the terrain (Sedgwick, 1986). First, the indoor consisted of a narrower hallway, compared with the outdoor pathway. Besides, in the indoor environment, there were doors and other objects on the sidewall, compared with only a few trees within the action space in the outdoor. Thus, the indoor environment provided a better linear perspective, which may have facilitated the midpoint estimations. Second, the heights of the terrestrial horizons in the two environments were different. The distance from the observer to the front wall indoor was 14.5 m, while the distance from the observer to the front building outdoor was 90 m. Previous studies have found that when linear perspective is fixed, raising the terrestrial horizon, results in an underestimation of the distance perception in VR (Messing & Durgin, 2005), as well as in the real world when the vision is degraded (Rand et al., 2011). Therefore, because the outdoor environment consisted of fewer linear perspective cues and higher heights of the terrestrial horizon in the current study, the estimated midpoints were more underestimated.

Previous studies have investigated the influence of environmental factors on distance estimation in the real world (Lappin et al., 2006; Philbeck et al., 2018; Witt et al., 2007) and VR (Bodenheimer et al., 2007). One study in particular (Lappin et al., 2006) found greater overestimations of the midpoints in real-world indoor (lobby and hall) compared with a real-world outdoor (lawn). In addition, some studies have shown that in long hallways, midpoint estimations are closer than those in shorter hallways (Witt et al., 2007), while others did not find a significant environmental effect on VR (Bodenheimer et al., 2007). Interestingly, our findings suggest a general underestimation of the midpoints in contrast to Lappin et al. (2006). Moreover, unlike Bodenheimer et al. (2007), we observed an effect of environmental context in VR. Perhaps factors such as the variable distance range of the midpoints and the sizes of targets may have contributed to these inconsistencies in the results across the studies. Therefore, further studies are required to investigate how these factors influence distance estimation.

In Experiment 2, the main finding of interest was that the mean walked distance in real-world outdoor was greater than that in real-world indoor, while the response consistency in real-world indoor was better than that in real-world outdoor. This may be influenced by two cognitive factors. The first factor is the top-down knowledge about the environmental context (Philbeck et al., 2018). When observers are outdoor, they know that there is an extended area, in which they can walk farther and are unlikely to bump into the walls or other obstacles. On the other hand, when the observers are indoors, the less extended area would limit the walking range, and they are more afraid to hit the sidewalls. Thus, observers tend to walk shorter indoors than outdoors. This is consistent with the restricted FOV condition in a previous blind-walking study (Philbeck et al., 2018). These findings are also in line with those in Iosa et al. (2012), which suggest that observers have more daily experience in walking in small indoor environments without vision. Thus, blind indoor walking is closer (suggested by the CEs) but more consistent (suggested by the VEs), because, in the dark, undershooting is safer than overshooting (Iosa et al., 2012). The second factor is the expectation of the distance range of the targets (Pagano & Isenhower, 2008). Even though in Experiment 2, the indoor distance range of the targets is identical to the outdoor distance range, it is still possible that observers have such an expectation that, due to the larger outdoor layout, the targets outdoor are expected to be further away than those in the indoor environment. Pagano and Isenhower (2008) have shown that the expectation of the distance range of the targets affects verbal distance estimates but not the rapid blind reaching. According to Goodale and Milner (1992), there are two separated neural pathways, a cognitive stream for conscious perception and a motor stream for action. It has been proposed that expectation only affects tasks that are processed through the cognitive stream but not those that are processed through the motor stream (Pagano & Isenhower, 2008). However, Experiment 2 showed that blind-walking was mediated by the expectation. There may be two explanations for this. First, there is also evidence suggesting that the two streams are responding to the unitary perceived distance (Philbeck & Loomis, 1997). Thus, if expectation meditates perceived distance, such a bias would potentially be observed in the outcomes from the motor stream as well. Second, unlike rapid blind reaching, blind-walking takes much more time and demands memory representation and updating during the walking (Loomis et al., 2002). This kind of procedure may require interaction with the cognitive stream, and thus, it is likely to be influenced by the expectation.

Notably, previous studies have already investigated the impact of the real-world environment on VR distance estimations (Interrante et al., 2006; Kelly et al., 2018). However, in these studies, only one real-world environment was used in the experiment, and the virtual environment was the replica of the real world. The current study, for the first time, demonstrated that a different real-world environment makes a difference to the distance estimated by blind-walking, but not to that estimated by bisection. Moreover, Experiments 2 and 3 showed that the virtual environment had no impact on the distance estimated by blind-walking. These findings are supported by an earlier study (Kelly et al., 2017), which showed a null effect of the virtual environment when blind-walking was compared across different virtual environments (virtual classroom and grass) with the same HMD as we used in the present study. It is also consistent with Kunz et al. (2009), which showed that the quality of graphics influences only for verbal reports, but not for blind-walking. Thus, it is possible that during blind-walking, unlike the bisection method, observers did not have any visual feedback from the virtual scene; hence, the representation of the virtual environment in the mind was weakened. As a result, the VR environmental context did not affect the walked distance.

In Experiment 2, we did not observe a general underestimation of distance perception as measured by blind-walking, possibly due to a practicing effect. However, in line with previous studies, the performance on Day 1 in Experiment 2 (a mean rate of 94.7% of the veridical distance), as well as the performance in Experiment 3 (a mean rate of 92.9% of the veridical distance), demonstrated a slight underestimation. The amount of underestimation in the current study was smaller than most of the previous studies, with a mean rate of 74% of the veridical distance (Renner et al., 2013). However, recent studies have demonstrated that with advanced HMDs, a distance estimated by blind-walking is more accurate and closer to that in the real world (Buck et al., 2018; Kelly et al., 2017; B. Li et al., 2016).

It should, however, be noted that the two measurement methods we used—bisection and blind-walking—are different in multiple ways. For instance, the shape and the distance of the targets differ between the two methods. Nevertheless, it is not within the scope of the present study to determine the exact factors that cause the differential effects of the environmental context. As a starting point, the current study intended to show that distance judgment in VR experience can be affected by not only the virtual environment but also by the real world. For now, we propose that one factor, that is, whether feedback was given when judgments were being made, may play an important role in distance judgment. When observers were adjusting the positions of the midpoints by bisection method, visual feedback from the virtual scene was given. On the contrary, during blind-walking, no feedback was available from the virtual scene, and perhaps the real-world surroundings may have restricted the walking action. Thus, the differential effect of environmental context was observed.

It should be noted that perceptual-motor calibration may play an important role in reducing the bias of distance perception in VR. Even in the real-world environment, it is common and necessary for us to coordinate actions with the feedback of our perception, to keep perceptual-motor systems accurate (e.g., Mon-Williams & Bingham, 2007). In the virtual environment, although the egocentric distance was generally underestimated, such distortion can also be calibrated through feedback and practice (Renner et al., 2013). For instance, it has been found that feedback with the estimated distance and actual distance (Richardson & Waller, 2005) improves the accuracy of distance estimates in VR. Other forms of feedback, such as auditory stop signal (Mohler et al., 2006) and free walking in the VR environment (Kelly et al., 2018), can also improve the distance estimates obtained by blind-walking. Thus, in Experiment 2 of the current study, it is possible that the effect of a real-world environment on blind-walking can also be eliminated by proper feedback. But we doubt that the feedback in Experiment 1 could calibrate the midpoint estimation because midpoint estimation depends only on visual perception and does not require motor responses (Bodenheimer et al., 2007), while blind-walking requires motor responses. As discussed earlier, conscious perception and vision-guided action may be processed through different pathways. Previous studies have only shown the effect of calibration in action but not perception. For instance, Altenhoff et al. (2012) have demonstrated that feedback improves blind reaching but not verbal estimates in VR. With a preview of the VR environment, blind-walking judgment was improved but size judgment was not (Kelly et al., 2018). Thus, although the role of the calibration was not tested in the current study, we hypothesize that perceptual-motor calibration would be effective in the blind-walking task but not in the bisection task. Further studies need to determine whether the bias caused by the virtual or real environment could be reduced by feedback and practice.

In conclusion, our findings demonstrate an environmental effect not only from the virtual scene but also from the real world, on distance estimation in VR. This is important to both theoretical studies and applications. First, this is the first study to show that real-world environment affects distance judgment measured by blind-walking in VR. This notwithstanding, further studies need to determine the exact cues in the real world that have an impact on the distance estimation in VR, especially when observers are not aware of the real-world surroundings, whether or not such unawareness would have a bias on the distance estimation in VR. Second, for the use of VR in training, veridical distance perception sometimes is crucial. To design such applications, the real-world surroundings and the users’ knowledge of the real world should also be taken into consideration. For instance, a limited room may bias the distance estimation when actions are performed in VR.

## References

[bibr1-20416695211023956] Altenhoff B. M. Napieralski P. E. Long L. O. Bertrand J. W. Pagano C. C. Babu S. V. Davis T. A. (2012). Effects of calibration to visual and haptic feedback on near-field depth perception in an immersive virtual environment. Proceedings, SAP 2012 - ACM Symposium on Applied Perception, 1, 71–78.

[bibr2-20416695211023956] Bian Z. Andersen G. J. (2013). Aging and the perception of egocentric distance. Psychology and Aging, 28(3), 813–825. 23276215 10.1037/a0030991 PMC3778099

[bibr3-20416695211023956] Bodenheimer B. Meng J. Wu H. Narasimham G. Rump B. McNamara T. P. , Carr, T. H., & Rieser J. J. (2007). *Distance estimation in virtual and real environments using bisection* [Paper presentation]. Proceedings of the 4th symposium on applied perception in graphics and visualization, July 26-27, Tubingen, Germany.

[bibr4-20416695211023956] Bruder G. Sanz F. Olivier A. Lecuyer A. (2015). *Distance estimation in large immersive projection systems, revisited* [Paper presentation]. 2015 Proceedings of IEEE virtual reality conference, March 23-27, Arles, France.

[bibr5-20416695211023956] Buck L. E. Young M. K. Bodenheimer B. (2018). A comparison of distance estimation in HMD-based virtual environments with different HMD-based conditions. ACM Transactions on Applied Perception, 15(3), 1–15.

[bibr6-20416695211023956] Creem-Regehr S. H. Stefanucci J. K. Thormpson W. B. Nash N. McCardell M. (2015). *Egocentric distance perception in the occulus rift (DK2)* [Paper presentation]. Proceedings of the ACM SIGGRAPH symposium on applied perception, September 13-14, Tübingen Germany.

[bibr7-20416695211023956] Cutting J. Vishton P. (1995). Perceiving layout: The integration, relative dominance, and contextual use of different information about depth. In Epstein W. Rogers S. (Eds.), Handbook of perception and cognition volume 5 perception of space and motion (pp. 69–117). Academic Press.

[bibr8-20416695211023956] Gajewski D. A. Philbeck J. W. Pothier S. Chichka D. (2010). From the most fleeting of glimpses: On the time course for the extraction of distance information. Psychological Science, 21(10), 1446–1453. 20732904 10.1177/0956797610381508 PMC3628738

[bibr9-20416695211023956] Gajewski D. A. Philbeck J. W. Wirtz P. W. Chichka D. (2014). Angular declination and the dynamic perception of egocentric distance. Journal of Experimental Psychology: Human Perception and Performance, 40(1), 361–377. 24099588 10.1037/a0034394 PMC4140626

[bibr10-20416695211023956] Geuss M. N. Stefanucci J. K. Creem-Regehr S. H. Thompson W. B. (2012). Effect of viewing plane on perceived distances in real and virtual environments. Journal of Experimental Psychology: Human Perception and Performance, 38(5), 1242–1253. 22409144 10.1037/a0027524

[bibr11-20416695211023956] Goodale M. A. Milner A. D. (1992). Separate visual pathways for perception and action. Trends in Neuroscience, 15(1), 20–25. 10.1016/0166-2236(92)90344-8 1374953

[bibr12-20416695211023956] Interrante V. Anderson L. Ries B. (2006, March). *Distance perception in immersive virtual environments, revisited* [Paper presentation]. IEEE Virtual Reality 2006, Proceedings, Alexandria, VA, United States.

[bibr13-20416695211023956] Iosa M. Fusco A. Morone G. Paolucci S. (2012). Walking there: Environmental influence on walking-distance estimation. Behavioural Brain Research, 226(1), 124–132. 10.1016/j.bbr.2011.09.007 21925542

[bibr14-20416695211023956] JASP Team. (2020). JASP (Version 0.14.1) [Computer software]. https://jasp-stats.org

[bibr15-20416695211023956] Kelly J. Cherep L. Siegel Z. (2017). Perceived Space in the HTC Vive. ACM Transactions on Applied Perception, 15(1), Article 2.

[bibr16-20416695211023956] Kelly J. W. Cherep L. A. Klesel B. Siegel Z. D. George S. (2018). Comparison of two methods for improving distance perception in virtual reality. ACM Transactions on Applied Perception, 15(2), 1–11.

[bibr17-20416695211023956] Kunz B. R. Creem-Regehr S. H. Thompson W. B. (2015). Testing the mechanisms underlying improved distance judgments in virtual environments. Perception, 44(4), 446–453 26492729 10.1068/p7929

[bibr18-20416695211023956] Kunz B. R. Wouters L. Smith D. Thompson W. B. Creem-Regehr S. H. (2009). Revisiting the effect of quality of graphics on distance judgments in virtual environments: A comparison of verbal reports and blind walking. Attention Perception & Psychophysics, 71, 1284–1293. 10.3758/APP.71.6.1284 19633344

[bibr19-20416695211023956] Lappin J. S. Shelton A. L. Rieser J. J. (2006). Environmental context influences visually perceived distance. Perception & Psychophysics, 68(4), 571–581. 16933422 10.3758/bf03208759

[bibr20-20416695211023956] Li B. Nordman A. Walker J. Scott K. (2016, July 22–23). *The effects of artificially reduced field of view and peripheral frame stimulation on distance judgments in HMDs* [Paper presentation]. Proceedings of the ACM Symposium on Applied Perception, Anaheim, CA, United States.

[bibr21-20416695211023956] Li Z. Phillips J. Durgin F. H. (2011). The underestimation of egocentric distance: Evidence from frontal matching methods. Attention, Perception & Psychophysics, 73(7), 2205–2217. 10.3758/s13414-011-0170-2 PMC3205207 21735313

[bibr22-20416695211023956] Livingston M. A. Ai Z. M. Swan J. E. Smallman H. S. (2009). Indoor vs. outdoor depth perception for mobile augmented reality. *IEEE Virtual Reality 2009, Proceedings*, 55–62.

[bibr23-20416695211023956] Loomis J. M. Knapp J. M. (2003). Visual perception of egocentric distance in real and virtual environments. In L. J. Hettinger & M. W. Haas (Eds.), *Virtual and adaptive environments* (pp. 21–46). Erlbaum.

[bibr24-20416695211023956] Loomis J. M. Lippa Y. Klatzky R. L. Golledge R. G. (2002). Spatial updating of locations specified by 3-D sound and spatial language. Journal of Experimental Psychology: Learning Memory and Cognition, 28, 335–345. 10.1037//0278-7393.28.2.335 11911388

[bibr25-20416695211023956] Loomis J. M. Philbeck J. (2008). Measuring spatial perception with spatial updating and action. In Klatzky R. L. MacWhinney B. Behrman M. (Eds.), Carnegie Mellon symposia on cognition. Embodiment, ego-space, and action (pp. 1–43). Psychology Press.

[bibr26-20416695211023956] Messing R. Durgin F. (2005). Distance perception and the visual horizon in head-mounted displays. ACM Transactions on Applied Perception, 2(3), 234–250.

[bibr500-20416695211023956] Mohler, B. J., Creem-Regehr, S. H. and Thompson, W. B. (2006) “The influence of feedback on egocentric distance judgments in real and virtual environments,” *Proc. – APGV 2006 Symp. Appl. Percept. Graph. Vis.*, no. January, pp. 93–100.

[bibr27-20416695211023956] Mon-Williams M. Bingham G. P. (2007). Calibrating reach distance to visual targets. Journal of Experimental Psychology: Human Perception and Performance, 33, 645–656. 17563227 10.1037/0096-1523.33.3.645

[bibr28-20416695211023956] Pagano C. C. Isenhower R. W. (2008). Expectation affects verbal judgments but not reaches to visually perceived egocentric distances. Psychonomic Bulletin & Review, 15, 437–442. 18488665 10.3758/pbr.15.2.437

[bibr29-20416695211023956] Philbeck J. W. Gajewski D. A. Jaidzeka S. M. Wallin C. P. (2018). The role of top-down. knowledge about environmental context in egocentric distance judgments. *Attention,* Perception, and Psychophysics, 80(2), 586–599 10.3758/s13414-017-1461-z 29204865

[bibr30-20416695211023956] Philbeck J. W. Loomis J. M. (1997). Comparison of two indicators of perceived egocentric distance under full-cue and reduced-cue conditions. Journal of Experimental Psychology: Human Perception and Performance, 23(1), 72–85. 9090147 10.1037//0096-1523.23.1.72

[bibr33-20416695211023956] Plumert J. M. Kearney J. K. Cremer J. F. Recker K. (2005). Distance perception in real and virtual environments. ACM Transactions on Applied Perception, 2(3), 216–233.

[bibr34-20416695211023956] Quintana D. S. Williams D. R. (2018). Bayesian alternatives for common null-hypothesis significance tests in psychiatry: A non-technical guide using JASP. BMC Psychiatry, 18, 1–8. 29879931 10.1186/s12888-018-1761-4 PMC5991426

[bibr35-20416695211023956] Rand K. M. Tarampi M. R. Creem-Regehr S. H. Thompson W. B. (2011). The importance of a visual horizon for distance judgments under severely degraded vision. Perception, 40(2), 143–154. 21650089 10.1068/p6843 PMC3315150

[bibr36-20416695211023956] Renner R. S. Velichkovsky B. M. Helmert J. R. (2013). The perception of egocentric distances in virtual environments – A review. ACM Computing Surveys, 46(2), 1–40.

[bibr37-20416695211023956] Richardson A. R. Waller D. (2005). The effect of feedback training on distance estimation in virtual environments. Applied Cognitive Psychology, 19, 1089–1108.

[bibr38-20416695211023956] Rieser J. Ashmead D. Talor C. Youngquist G. (1990). Visual perception and the guidance of locomotion without vision to previously seen targets. Perception, 19(5), 675–689. 2103000 10.1068/p190675

[bibr39-20416695211023956] Sedgwick H. A. (1986). Space perception. In Kaufman B. Thomas J. (Eds.), Handbook of perception and human performance: Vol. 1. Sensory processes and perception. Wiley-Interscience.

[bibr40-20416695211023956] Sinai M. J. Ooi T. L. He Z. J. (1998). Terrain influences the accurate judgement of distance. Nature, 395(6701), 497–500. 9774104 10.1038/26747

[bibr41-20416695211023956] Witt J. K. Stefanucci J. K. Riener C. R. Proffitt D. R. (2007). Seeing beyond the target: Environmental context affects distance perception. Perception, 36(12), 1752–1768. 18283926 10.1068/p5617

[bibr42-20416695211023956] Wu B. Ooi T. L. He Z. J. (2004). Perceiving distance accurately by a directional process of integrating ground information. Nature, 428(6978), 73–77. 14999282 10.1038/nature02350

